# Acute flaccid paralysis of a new surfer: A case report

**DOI:** 10.1097/MD.0000000000029188

**Published:** 2022-04-22

**Authors:** Yee Leng Loh, Sridhar Atresh, Kylie Ferguson

**Affiliations:** Princess Alexandra Hospital, Woolloongabba Queensland, Australia.

**Keywords:** inpatient rehabilitation, multidisciplinary/interdisciplinary rehabilitation, neurological disorders, spinal cord injury, surfer's myelopathy

## Abstract

**Rationale:**

Surfer's myelopathy is a rare atraumatic spinal cord injury most frequently experienced by novice surfers. Patients often experience back pain, followed by motor, sensory, bowel, and bladder involvement. Here, we report a case of surfer's myelopathy.

**Patient concerns:**

The patient presented with acute low back pain associated with lower limb weakness, sensory loss, urinary retention, and perineal paraesthesia 1 hour after her first surf lesson.

**Diagnosis:**

On arrival at the emergency department, she was noted to have flaccid paralysis with flickers in both lower limbs, reduced sensation in the midthoracic region, reduced anal tone, and saddle anesthesia. Magnetic resonance imaging of the spine revealed evidence of restricted diffusion from T6 to the level of the conus. Extensive investigations, including cerebrospinal fluid analysis, vasculitides/paraneoplastic screening, and further imaging, were unremarkable. She was diagnosed with complete T7 spinal cord injury secondary to surfer's myelopathy.

**Interventions:**

She subsequently received methylprednisolone and was transferred to the spinal injury unit for rehabilitation. As she experienced persistent neuropathic pain at the level of the injury, she received input from the local pain team. One month after the injury, the patient developed swelling of the right thigh associated with reduced internal and external rotation of the right hip, impacting rehabilitation. The patient was diagnosed with heterotopic ossification following a triple-phase bone scan. She then received intravenous zolendronic acid, which had a good effect.

**Outcomes:**

Four months after the initial presentation, she was discharged to the community. Despite no improvement in her neurological status, she was independent of transfers and mobility with a wheelchair. In addition, she managed her neurogenic bowel and bladder independently with intermittent self-catheterization and a transanal irrigation system. At 6 months, she engaged well with returning to drive program and vocational rehabilitation.

**Lessons:**

Neurological recovery from surfer's myelopathy has been shown to vary from complete recovery to minimal recovery. With a spinal-specific rehabilitation program, this patient remains independent of her activities of daily living. Surfer's myelopathy often occurs in inexperienced surfers; therefore, it is crucial to provide education to surfers and instructors.

## 1 Introduction

Surfer's myelopathy is a rare atraumatic spinal cord injury most frequently experienced by novice surfers.^[1–4]^ The condition was first recorded by Thompson et al^[5]^ in 2004 with 9 cases identified between 1998 and 2003. Patients often experience back pain followed by motor, sensory, bowel, and bladder involvement.^[6]^ Postulated risk factors included novice surfing ability, thin physique, poor back muscle strength, dehydration, significant Valsalva maneuver, deep vein thrombosis related to long flights, central canal stenosis, spondylolisthesis, and extended hypotension.^[1,5]^ We report the case of a 35-year-old woman who presented with acute low back pain, paralysis, and urinary retention after her first surf lesson.

## 2 Case presentation

A 35-year-old woman presented to the hospital with acute low back pain associated with lower limb weakness, sensory loss, urinary retention, and perineal paraesthesia 1 hour after her first surf lesson. On arrival at the emergency department, she was noted to have flaccid paralysis with flickers in both lower limbs, reduced sensation in the midthoracic region, reduced anal tone, and saddle anesthesia. The patient was systemically well prior to the event. There was no history of underlying autoimmune disease. Her family members were healthy.

**Figure 1:**
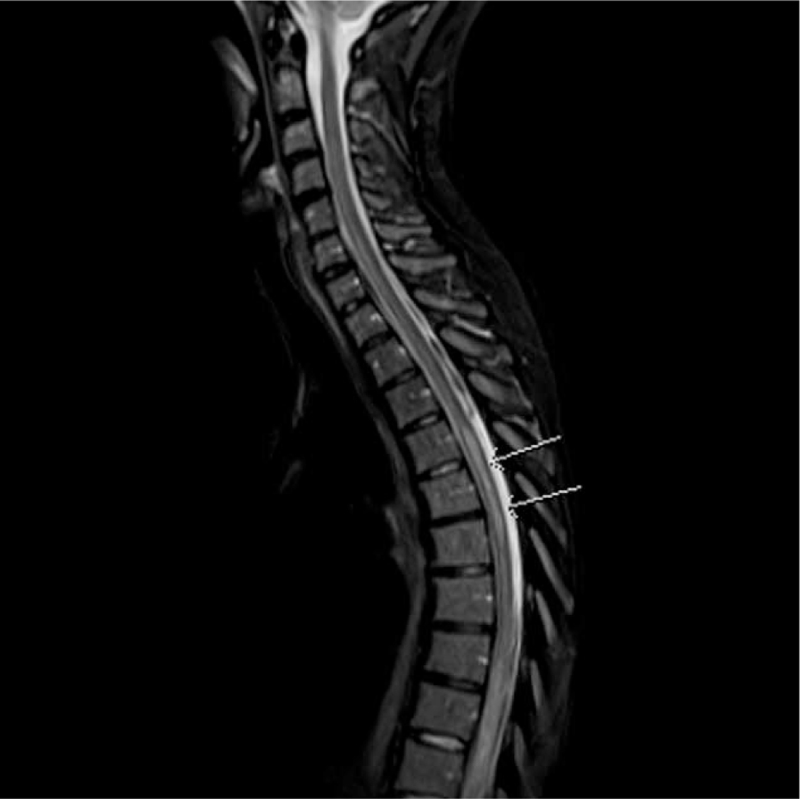
Sagittal STIR image of MRI spine demonstrated increase signal from midthoracic extending to the level of conus medullaris. MRI = magnetic resonance imaging.

**Figure 2:**
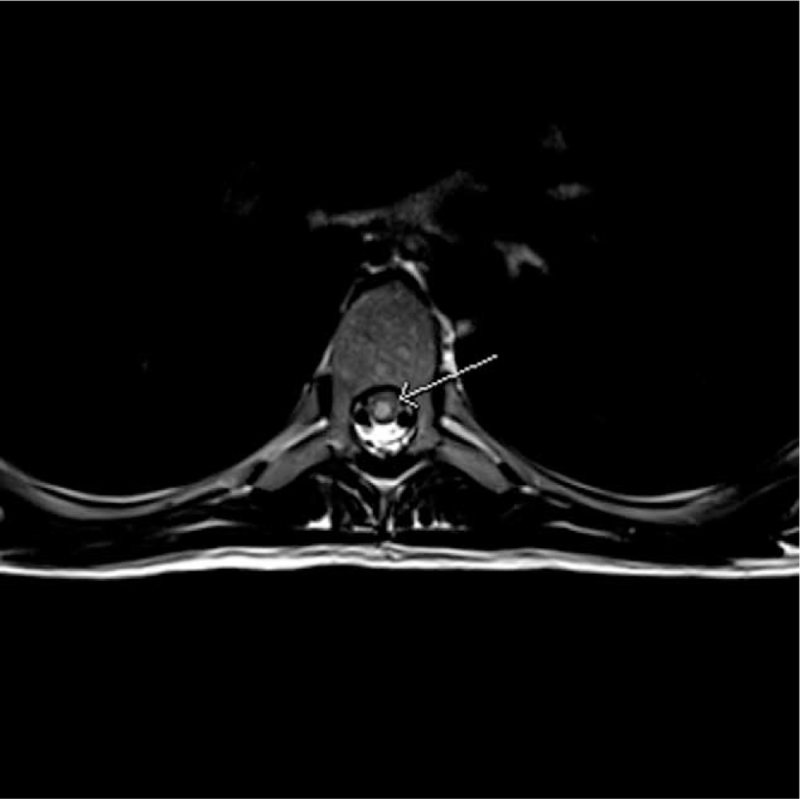
Axial T2 sequence image of MRI spine demonstrated increase signal in central cord at T6 level. MRI = magnetic resonance imaging.

She underwent lumbar puncture, which showed an opening pressure of 11 cm H_2_O, normal biochemical study, negative cerebrospinal fluid culture, viral studies, antineuromyelitis optica antibodies, and lymphoproliferative markers. Serological examination was negative for systemic vasculitides and paraneoplastic antibodies. Magnetic resonance imaging (MRI) of the spine showed evidence of hyperintensity on the T2 sequence from T6 down to the level of the conus (Figs. 1 and 2), with evidence of restricted diffusion. The postcontrast sequences were unremarkable with no evidence of cord enhancement, mass lesions, or large vessel vasculitis. She subsequently received a course of methylprednisolone. A computed tomography angiogram of the spine showed no vascular abnormalities. She was then diagnosed with T7 AIS A spinal cord injury spinal cord injury secondary to surfer's myelopathy based on the clinical correlation of the novice surfer with MRI findings and negative serologic studies.

**Figure 3:**
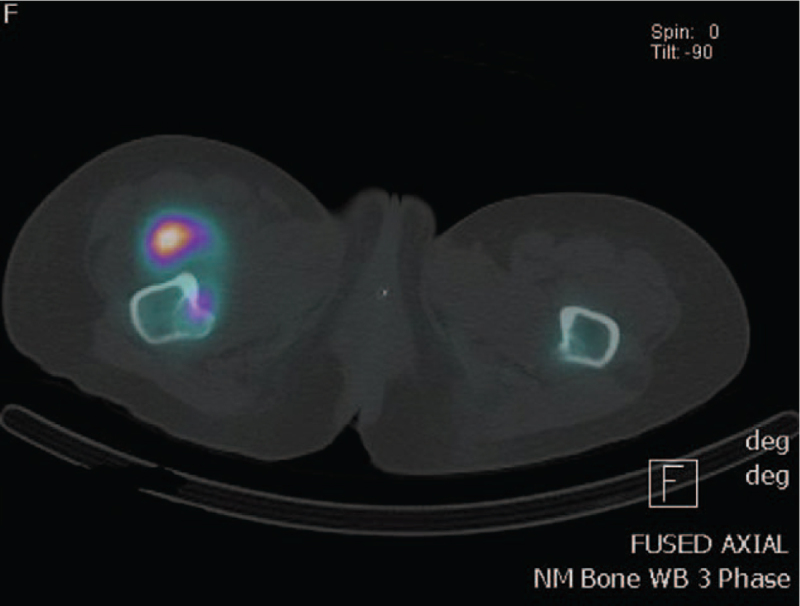
Tripple phase bone scan showed a focal region of mild to moderate hyperaemia and moderate to intensely increased delayed radiotracer uptake in the anteromedial aspect of the proximal right thigh. This is in keeping with a site of active heterotopic ossification.

**Figure 4:**
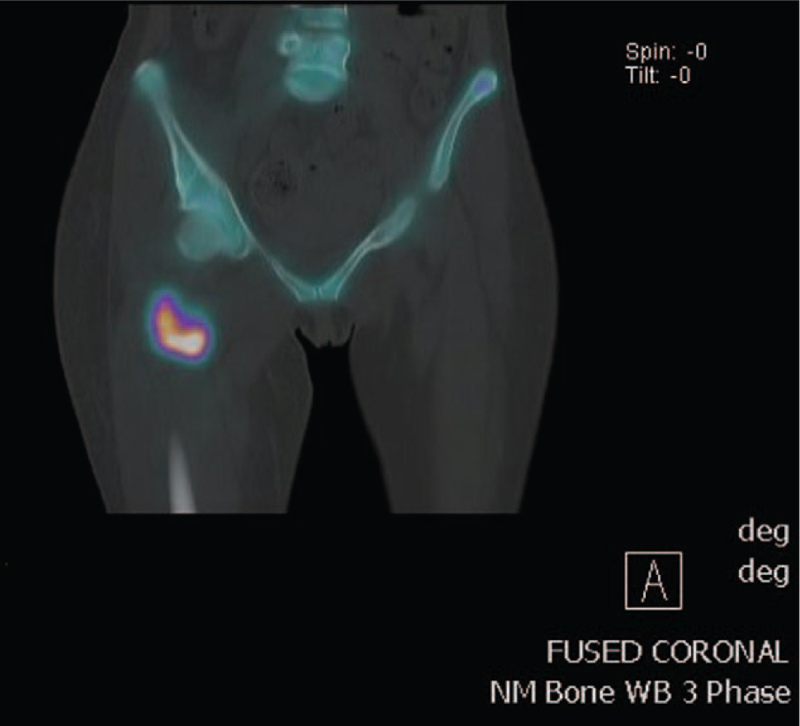
Tripple phase bone scan showed a focal region of mild to moderate hyperaemia and moderate to intensely increased delayed radiotracer uptake in the anteromedial aspect of the proximal right thigh. This is in keeping with a site of active heterotopic ossification.

Persistent neuropathic pain remains a significant issue in rehabilitation. She underwent a multifaceted approach to manage her ongoing pain. Ketamine and lignocaine infusions were trialled with minimal effect, and she could not tolerate duloxetine. A combination of lignocaine patch at the thoracic level, gabapentin, buprenorphine patch, mianserin, and topical menthol salicylate cream managed to bring the pain to a manageable level. Nonpharmacological interventions, such as mindfulness, distraction, deep breathing exercises, and good sleep hygiene, were as effective as medication. One month following the injury, the patient developed swelling of the right thigh associated with reduced internal and external rotation of the right hip. She was diagnosed with heterotopic ossification following a triple-phase bone scan (Figs. 3 and 4). Her right hip range of motion improved with intravenous zolendronic acid and intensive physical therapies.

Four months after the initial presentation and completing her rehabilitation programme, the patient was discharged to the community. Despite no improvement in neurological status, she was independent of her transfer and mobility with wheelchair. She was able to negotiate ascending/descending ramps and balance the back wheels of her manual wheelchair. She was independent of her self-care, including managing her neurogenic bladder with intermittent clean self-catheterization and Peristeen transanal irrigation twice daily for her neurogenic bowel. She was linked with the local rehabilitation team, the Transition Rehabilitation Program, and the Spinal Outreach Team to facilitate community integration. At 6 months, she engaged well with the return to driving program and vocational rehabilitation.

## 3 Discussion

The mechanism underlying this condition remains unknown. One postulated cause is ischemic injury of the lower thoracic spine, thought to be secondary to a continuous hyperextension posture, which contributes to blood flow insufficiency in the region.^[4,5,7]^ Other possible causes include fibrocartilaginous embolism, possibly secondary to the Valsalva maneuver, or venous infarction due to inferior vena cava obstruction of the liver, which is thought to be secondary to the hyperextended position on the surfboard.^[3,6]^

Spinal magnetic resonance imaging often shows a T2 hyperintense lesion in the thoracic region, with conus involvement in some cases.^[2,4]^ Spinal angiograms have been recommended for the workup of surfer's myelopathy.^[8]^

Acute intervention recommendations include hydration, induced hypertension, and corticosteroids, although there is currently no consensus regarding treatment.^[3]^ In a study by Chang et al^[8]^ (2012), they found that treatment with methylprednisolone did not have significant outcomes, and the mean arterial pressure did not correlate with outcomes. The most effective intervention was intensive multidisciplinary rehabilitation.^[3,9,10]^ Neurological recovery has been shown to vary from complete recovery to minimal recovery.^[2]^

## 4 Conclusion

With a spinal-specific rehabilitation program, this patient remains independent of her activities of daily living and integrated well into the local community. As surfer's myelopathy often occurs in inexperienced surfers, it is crucial to provide education to surfers and instructors. There is a mnemonic on the Surfer's Myelopathy Foundation website to help reduce the risk of surfer's myelopathy.^[6]^

SPINE

“S sit on your board while waiting for waves

P pace your time in the water (30-minute limit)

I insist on a knowledgeable surfing instructor

N notice signs of pain and discomfort in your back

E exit the water and seek medical attention if you experience pain, tingling, or weakness”.^[6]^

## Acknowledgments

We wish to thank Dr. Paul Schmidt for his assistance in reporting the MRI findings and the team members involved in the care of this patient.

## Author contributions

**Conceptualization:** Loh Yee Leng, Atresh Sridhar

**Data curation:** Loh Yee Leng, Atresh Sridhar, Ferguson Kylie

**Formal analysis:** Loh Yee Leng, Atresh Sridhar

**Funding acquisition:** Loh Yee Leng, Atresh Sridhar

**Methodology:** Loh Yee Leng, Atresh Sridhar

**Resources:** Loh Yee Leng, Atresh Sridhar, Ferguson Kylie

**Supervision:** Atresh Sridhar

**Writing – original draft:** Loh Yee Leng, Ferguson Kylie

**Writing – review & editing:** Loh Yee Leng
